# Vulvar Lichen Sclerosus: Navigating Sex Hormone Dynamics and Pioneering Personalized Treatment Paradigm

**DOI:** 10.3390/jpm14010076

**Published:** 2024-01-08

**Authors:** Adelina Popa, Mihai Cristian Dumitrascu, Aida Petca, Razvan-Cosmin Petca, Florica Sandru

**Affiliations:** 1 Department of Dermatovenerology, “Carol Davila” University of Medicine and Pharmacy, 020021 Bucharest, Romania; adelina.popa@drd.umfcd.ro (A.P.); florica.sandru@umfcd.ro (F.S.); 2 Dermatology Department, “Elias” University Emergency Hospital, 011461 Bucharest, Romania; 3 Department of Obstetrics and Gynecology, “Carol Davila” University of Medicine and Pharmacy, 020021 Bucharest, Romania; aida.petca@umfcd.ro; 4 Department of Obstetrics and Gynecology, University Emergency Hospital of Bucharest, 050098 Bucharest, Romania; 5 Department of Obstetrics and Gynecology, “Elias” Emergency University Hospital, 011461 Bucharest, Romania; 6 Department of Urology, “Carol Davila” University of Medicine and Pharmacy, 020021 Bucharest, Romania; razvan.petca@umfcd.ro; 7 Department of Urology, ‘Prof. Dr. Th. Burghele’ Clinical Hospital, 050659 Bucharest, Romania

**Keywords:** vulvar lichen sclerosus, puberty, photodynamic therapy, carbon dioxide laser, clobetasol propionate, progesterone, platelet-rich plasma, stem cell therapy

## Abstract

Vulvar lichen sclerosus (VLS) is a frequently overlooked inflammatory disorder affecting the skin and mucous membranes of the vulva. With a propensity for atrophy, severe scarring, functional impairment, and malignant evolution, VLS is a disease that recurs frequently; early diagnosis, rapid treatment, and ongoing patient follow-up are essential. Potent topical corticosteroids (TCSs) are now widely recognized as the most effective treatment for achieving remission in VLS, but considering the potential complications of long-term treatment with potent TCSs, understanding the evolution of VLS during puberty becomes particularly crucial in determining the necessity for aggressive or more conservative therapeutic interventions. Emerging treatments, including PRP (platelet-rich plasma), stem cell therapy, and energy-based lasers like fractional CO_2_ and Nd-YAG, are being investigated to identify more effective VLS treatments than ultrapotent topical corticosteroids. However, more research is needed to assess the efficacy and safety of these new medicines. Topical clobetasol 0.05% ointment daily for 4–12 weeks is the gold standard for treating VLS. This article is a narrative review of the English-language medical literature from 2017 to November 2023, following three main sections concerning VLS: studies of the evolution amid pubertal hormonal changes; studies of the outcomes of personalized conventional therapies; and studies addressing the spectrum of innovative modalities for VLS.

## 1. Introduction

Vulvar lichen sclerosus (VLS) is a frequently overlooked inflammatory disorder affecting the skin and mucous membranes of the vulva and was initially described in 1887 [1]. Since then, a number of synonyms have been used, including “guttate scleroderma”, “lichen sclerosus et atrophicus”, “white spot disease”, “vulvar dystrophy”, and “Kraurosis vulvae”. The word “lichen sclerosus”, now used for both genital and extragenital lesions, has superseded all of these other designations [1,2].

VLS produces chronic itching and pain in the vaginal area and the area surrounding the anus. Scarring following inflammation can cause serious harm by causing the clitoris to be buried in women and girls, the vulval lips (labia) to fuse, and the vaginal aperture to constrict; with a propensity for atrophy, severe scarring, functional impairment, and malignant evolution, VLS is a disease that recurs frequently [3,4]. Early diagnosis, rapid treatment, and ongoing patient follow-up are essential [5]. Spontaneous remissions rarely occur [4]. Although no cure for LS exists, it can be managed with proper care [4]. Early intervention can potentially avert long-term consequences such as anatomical structural deterioration and the development of squamous cell carcinoma (SCC) [6]. Controversies surrounding the pathogenesis of VLS have prompted extensive research, leading to the exploration of various treatment modalities. Consequently, our attention has been meticulously directed towards a thorough review of the existing literature with the aim of incorporating an up-to-date perspective on the management of this pathology. In that order, dermatologists, gynecologists, urologists, histopathologists, surgeons, general practitioners, and pediatricians must have an accurate understanding of the condition and be willing to collaborate if necessary on behalf of the affected patients [7,8,9].

### 1.1. Unraveling the Complexities: Exploring the Role of Sex Hormones in Vulvar Lichen Sclerosus

Due to the shortage of comprehensive epidemiologic screening studies in this field and a sizable portion of cases may be asymptomatic (15–40%), the actual prevalence of LS is unknown [10,11,12]. Previous research has revealed LS to be an uncommon illness that affects 1 in 300–1000 women [13]; nevertheless, more recent research indicates a greater frequency (1 in 60) [14], and it can potentially reach as high as 1 in 30 in older populations [15]. According to a research study conducted in the United States from 2015 to 2017, LS had a claims-based prevalence of 0.05%, which is lower than previously reported and shows considerable underdiagnosis [16]. Although VLS affects women of all ages, its prevalence peaks at two different ages: prepubertal girls (1 in 900) and peri- or post-menopausal women (i.e., after menopause) [17,18]. These peaks support the idea that hypoestrogenism plays a role in the development of VLS. The first peak is expected to comprise 7–15% of all cases [19]. Nevertheless, not all authors find it consistent [10,14]. The range of the mean age at diagnosis is 52.6 to 60 years old [10,14,20,21,22]; however, the length of time symptoms can last before that can be substantial (68 ± 11.2 months) [10], raising doubts about the actual role of menopause in the disease’s development.

A possible explanation for its genesis is the vulva’s decreased level of the enzyme 5α-reductase. In a study, 30 women with untreated VLS had their serum hormone levels (estradiol, testosterone, dihydrotestosterone, androstenedione, and sex hormone-binding globulin) measured. Patients with untreated VLS had significantly higher levels of free testosterone and considerably lower levels of dihydrotestosterone and androstenedione in their serum when compared to typical values for their age [23]. Additionally, a number of studies have shown that VLS has fewer nuclear androgen receptors and that well-developed VLS has fewer androgen receptors than early VLS [24,25,26]. As proposed by Clifton et al., the loss of androgen receptor expression (down-regulation) in VLS may be secondary, resulting from a change in the squamous phenotype rather than a hormonal etiology [24].

The change from vaginal to vulva genitalia in a normal female is characterized by an increase in androgen receptors and a decrease in estrogen and progesterone receptors. A subset of LS patients appears to have lower vulvar androgen receptor expression [24,25]. Recent research indicates that oral contraceptive pills, particularly those with antiandrogenic qualities, may disrupt the androgen-dependent growth of the vulvar skin, thereby causing the early onset of LS in a minority of sensitive young women [27,28].

### 1.2. Interplay of Autoimmunity and Genetics in VLS

Research has documented a significant prevalence of autoimmune disease in individuals with VLS, along with much greater levels of autoantibody detection; however, it is essential to note that this does not definitively establish VLS as an autoimmune condition [29,30]. A critical complicating element is that middle-aged female patients, who make up the primary group affected by this disorder, exhibit a considerably elevated prevalence of autoantibodies [30]. A meticulously conducted study revealed that individuals with VLS experience autoimmune disease at a higher frequency compared to individuals of the same age who do not have VLS; approximately 30% of VLS patients are affected by autoimmune disease, whereas the prevalence in the general population is 10%. In addition, about 30% of individuals had a favorable familial background. Nevertheless, the investigation revealed no notable disparity in the rate of autoantibody detection between individuals with VLS and the control group. The two disorders most frequently observed in conjunction were autoimmune thyroid disease and vitiligo [31]. Furthermore, VLS has been associated with morphea, alopecia areata, and pernicious anemia [32,33]. There have been reports of diabetes, psoriasis, and celiac disease co-existing; however, this may be a coincidence [34,35,36].

A frequently cited study found that 67% of the 30 patients with LS in the group had serum immunoglobulin G (IgG) antibodies to ECM-1, while only 7% of the control group had these antibodies [37]. The study, regrettably, has not provided us with any additional understanding of the etiology. Nevertheless, it does reinforce the assumption that VLS is an autoimmune disorder, even if this hypothesis has not been verified [37,38]. Encountering a low-titer positive antinuclear antibody (ANA) is not unusual, although it is seldom important enough to justify more research [39]. If thyroid autoantibodies are detected, additional examination is necessary [40,41]. Despite the existence of thyroid autoantibodies, thyroid function can remain within the normal parameters [41,42].

Familial occurrence of LS is observed; however, it is predominantly attributed to random chance [43,44]. Consequently, there has been a quest to identify a correlation between human leukocyte antigen (HLA) and the situation [43]. While there have been no observed associations with the autoimmune-related HLA antigens HLA A1, B8, and DR3, the HLA class II antigens HLA-DQ7, HLA-DR11, and HLA-DR12 have the highest susceptibility to LS [45]. Although the confirmed HLA connections are intriguing, insufficient substantial data can definitively evaluate these associations’ strength [46].

Epigenetic modifications can lead to functional deficits in the genome unrelated to the DNA sequence. These modifications can result in modified gene expression and phenotypic alterations. A recent study has discovered changes in the enzyme expression in VLS caused by an epigenetic alteration. This finding suggests that there may be an epigenetic basis for developing the disease [47].

### 1.3. VLS: Current Treatment Paradigm

Potent topical corticosteroids (TCSs) are now widely recognized as the most effective treatment for remission in VLS [48,49]. The initial documentation of this therapy was released in 1991, utilizing clobetasol propionate (CP) 0.05%, a powerful TCS [50]. Subsequently, numerous further reports were published [51,52,53]. Prior to the publication of that article [50], it was deemed inconceivable to administer potent TCSs to the skin of the genitals. Instead, treatment protocols involved the administration of mild TCSs, as well as testosterone and progesterone; consequently, VLS was deemed highly challenging to cure [54,55].

Studies and publications commonly indicate that the problem cannot be resolved independently and requires management. However, there is no agreement on the specific methods for long-term treatment of the condition [56,57,58]. The release of a recent guideline acknowledged that treatment for women is still inadequate, as scar development, which can cause disability, is a common occurrence despite treatment. According to the same evaluation, the treatment primarily focuses on reducing symptoms, and proactive management may be considered to sustain remission in cases of active disease. Still, no explicit suggestion was provided [57].

Tacrolimus and pimecrolimus, topical immunosuppressive medications, may treat VLS in children and adults [50,59,60,61]. In a phase II trial from 2006, tacrolimus ointment 0.1% was tested for VLS patients; at 24 weeks, 43% of patients had total active LS resolution and 34% had partial resolution [62]. Topical immunosuppressive medicines may increase the risk of malignant transformation, even though no adverse events occurred throughout the 18-month monitoring period. Pimecrolimus therapy can cause SCC in VLS patients [63].

Topical tretinoin has been utilized as the sole treatment for VLS. Still, insufficient evidence supports its effectiveness, and it may be restricted due to its potential to cause irritation [64].

Phototherapy (narrowband UVB, UVA1, and topical PUVA phototherapy) [65,66,67] for VLS should only be considered after traditional therapies have proven ineffective, as it does not offer superior symptom alleviation, quality of life improvement, or practicality compared to TCSs [68]. In addition, the occurrence of skin cancer following phototherapy can be problematic in the genital areas, especially considering the heightened risk of cancer in VLS [69]. Photodynamic therapy (PDT) using topical 5-aminolevulinic acid (5-ALA) is a viable treatment option for uncontrollable itching in VLS when other treatments have been unsuccessful [68]. Nevertheless, there is disagreement regarding the clinical and histological improvements [70]. Some instances showed healing of superficial erosions and improvement of clinical symptoms after PDT, while others did not show any clinical changes [71,72]. However, other researchers have reported an increased occurrence of apoptosis and the eradication of persistent inflammation [73,74]. Surgery for women with anogenital LS should only be considered for patients who have vulvar intraepithelial neoplasia or malignancy or for those who need correction of scarring that is affecting normal function. Introital stenosis can cause problems with urination or sexual intercourse and may necessitate introital widening. It is advisable to postpone surgery until the disease activity has subsided [1].

### 1.4. Aim

We aim to assess recent advances in treatment research and to correlate pubertal evolution with treatment needs in VLS by addressing past literature gaps.

## 2. Methods

This is a narrative review of the English-language medical literature, covering six years of the most recent publications (from 2017 to November 2023) in which we included 25 original papers concerning VLS and innovative therapeutic approaches, identified through a PubMed-based search, with the following combinations of keywords: “vulvar lichen sclerosus” and “menarche” or “puberty”, “pediatric”, “sex hormones”, “treatment” or “progesterone”, “testosterone”, “stem cell”, “carbon dioxide laser”, “photodynamic”.

Exclusion criteria were case reports or case series; LS and its complications in men; surgical treatment modalities for VLS complications; and evaluation of participating girls limited to pre-pubertal assessment in the study.

**Figure 1:**
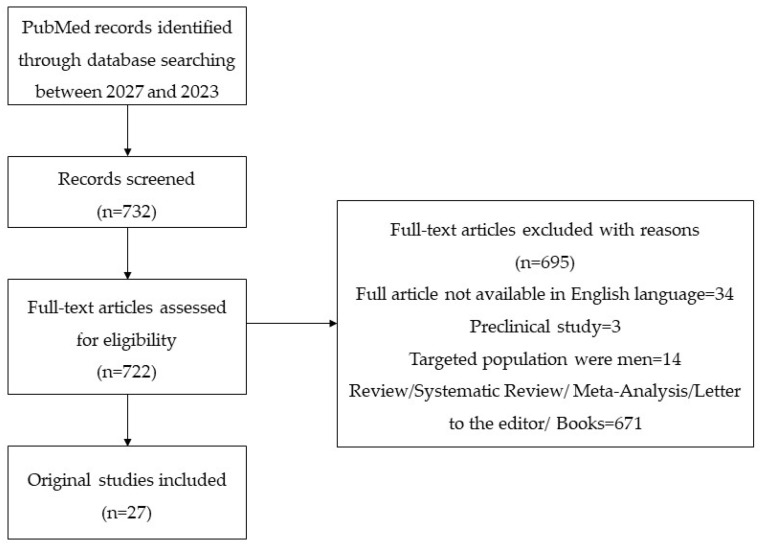
PRISMA flow diagram of the screening process.

We followed three main sections concerning VLS: studies of the evolution amid pubertal hormonal changes, studies of the outcomes of personalized conventional therapies, and studies addressing the spectrum of innovative modalities for VLS, including platelet-rich plasma and stem cell therapies. A PRISMA flow diagram was created in order to visually summarise the screening process (Figure 1).

## 3. Results

### 3.1. Dynamics of Sexual Hormones throughout Puberty and Beyond in VLS

VLS is most common in women of prepubertal and postmenopausal age groups, suggesting hormonal considerations may contribute to its origin. Although topical testosterone treatments have been popular for treating LS, subsequent controlled trials have shown ineffectiveness, casting doubt on any causal relationship with androgens. Whether alterations in estrogen metabolism contribute to LS is unclear; however, topical and systemic estrogens have been found to be effective treatments during periods of relative estrogen insufficiency [17]. Pediatric VLS (pVLS) accounts for 10–15% of all instances of VLS, and it is believed to affect approximately 1 in every 900 girls before they reach puberty [75]. Nevertheless, this is likely a conservative estimation, as initial indications and symptoms are typically vague, and the diagnosis may go unnoticed. In their study, Lagerstedt et al. assert that a mere 16% of individuals with VLS receive an early diagnosis of the disease [69,76,77,78]. According to Morrel et al.’s recent research, the remission rates showed significant variation, ranging from 11% to 70%. While there is disagreement among clinicians over the exact rates of remission and progression of the disease, they now recognize that pVLS can continue beyond puberty [79]. Considering the potential complications of long-term treatment with potent TCSs, understanding the evolution of VLS during puberty becomes particularly crucial in determining the necessity for aggressive or more conservative therapeutic interventions.

Winfrey OK et al. conducted a retrospective study to determine the frequency of persistent vulvar LS during the pubertal transition and to assess potential differences in symptomatology and examination findings based on menarchal status at the onset of symptoms. The study included 196 females aged 21 years or younger diagnosed with VLS. Among these patients, 141 were premenarchal, and 55 were postmenarchal, with 36 having symptoms onset postmenarche and others having premenarchal symptom onset or VLS diagnosis. During the data review period, 26 patients were followed through the pubertal transition, and 10 (38.5%) continued to experience LS symptoms. The premenarchal group (N = 141) was significantly more likely than the symptom-onset postmenarchal group (N = 36) to present with vulvar itching (70.2% vs. 52.8%; *p* = 0.048), vulvar bleeding (26.2% vs. 5.6%; *p* = 0.008), and bowel symptoms (16.3% vs. 0%; *p* = 0.009). On examination, the premenarchal group was significantly more likely to have subepithelial hemorrhages (24.8% vs. 5.6%; *p* = 0.01). In contrast, the postmenarchal group exhibited more clitoral adhesions (25.0% vs. 4.3%; *p* < 0.0001) and loss of labia minora (47.2% vs. 2.1%; *p* < 0.0001). Thirteen postmenarchal patients reported dyspareunia. This study suggests that premenarchal VLS can persist after menarche in approximately 40% of adolescents. It may also initially develop in postmenarchal adolescents, with differences in initial symptoms and examination findings based on menarchal status [80] (Table 1).

In a study conducted in a single center, 31 patients with pVLS with a mean age at diagnosis of 6.3 years (SD ± 2.58), with 22% having autoimmune comorbidities, and 19.4% having a family history of genital or extragenital LS were reached for re-evaluation in adulthood (mean follow-up time of 14 years, SD ± 6.9), when the mean age was 20.3 years (SD ± 6.68). Among them, 25.8% had received a second autoimmune disease diagnosis (seven thyroid diseases and one celiac disease). Histological confirmation of VLS at re-examination was observed in 12.9% of cases, while 61.3% reported no relevant symptoms. Symptoms reported included itching (12.9%), burning (12.9%), vulvar discomfort (12.9%), dyspareunia (6.5%), and vulvar dryness (9.7%). The mean age at symptom resolution was 13 years (SD ± 2.9), and the mean age at menarche was 13.4 years (SD ± 0.8). Upon follow-up, 58.1% were still affected by VLS, 16.1% achieved complete remission, and 25.8% were asymptomatic despite clinical signs. Analysis of factors potentially associated with persistent pVLS post-menarche showed no statistically significant associations, with relative risks (RR) of 0.8 (CI 0.6–1) for steroid prescription at diagnosis, 1.4 (CI 1.0–1.8) for comorbid autoimmune diseases, and 1.4 (CI 1.0–1.8) for familial VLS [81].

### 3.2. VLS: Insights into Conventional Therapeutic Approaches

**Figure 2:**
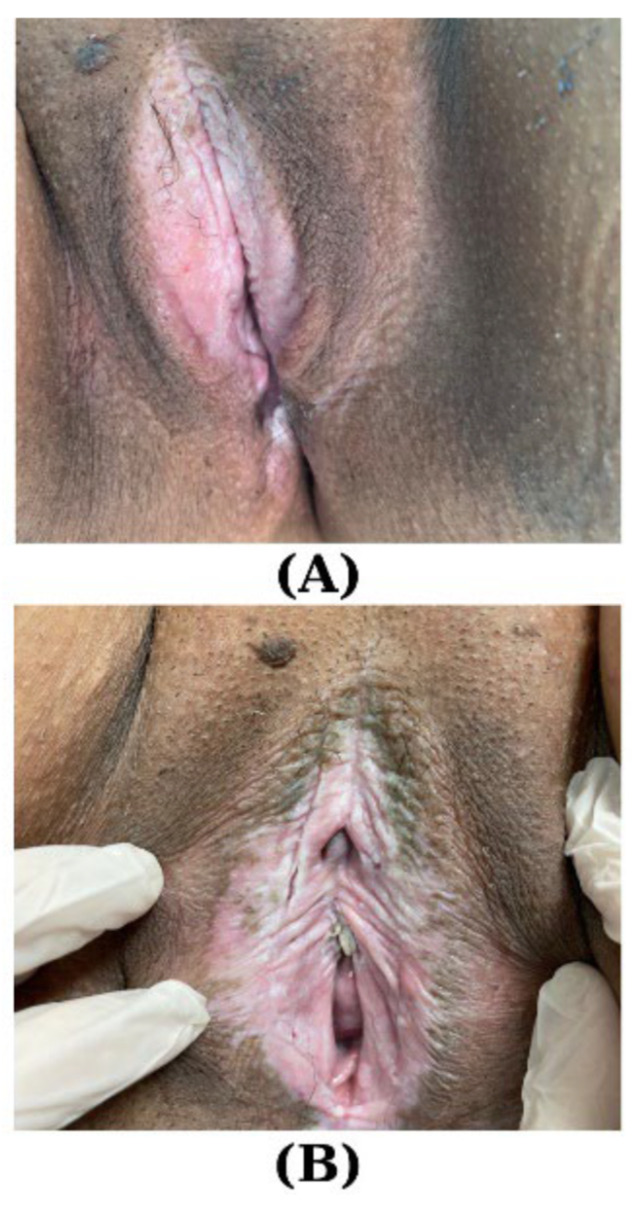
(**A**) A 58-year-old female, who presented with VLS, presents atrophy, hypopigmentation, and loss of vulvar architecture; (**B**) image of VLS lesions in the same patient after 12 weeks of topical CP—slight improvement in clinical appearance but significant enhancement in symptomatology.

In a retrospective observational study involving 62 cases of VLS and 86 cases of other dermatoses of the vulvar area, the age comparison revealed a statistically significant difference (*p* = 0.002), with individuals afflicted by VLS exhibiting an average age of 46.33 ± 2.33 years, compared to 41.01 ± 1.29 years in the other dermatoses group. The treatment patterns displayed compelling variations. The use of ultra-potent TCSs was remarkably higher in the VLS cohort, with 72.6% receiving this intervention compared to 18.6% in the other dermatoses group (*p* < 0.001). Additionally, topical calcineurin inhibitors were exclusively employed in VLS cases (9.7% vs. 0%, *p* = 0.005). Pelvic floor physiotherapy, on the other hand, was more prevalent among patients with other dermatoses (29.1% vs. 14.5%, *p* = 0.038). These findings underscore the nuanced treatment landscape for these dermatological conditions, providing valuable insights for clinicians and researchers alike. Furthermore, adjuvant treatments, such as topical ketoconazole, demonstrated significant disparities between the two groups, emphasizing the need for tailored therapeutic approaches in managing VLS [82] (Figure 2).

In a recent randomized, double-blinded, two-arm, single-center study involving 37 premenopausal women with histologically confirmed VLS, the efficacy of topical progesterone 8% ointment was evaluated and compared to standard therapy with topical CP 0.05%. The participants were randomized in a 1:1 ratio, with 17 receiving progesterone and 20 receiving CP. After 12 weeks, the mean clinical LS scores showed improvement from 4.6 (SD 2.0) to 4.5 (SD 1.7) in the progesterone arm and from 4.6 (SD 2.8) to 2.9 (SD 2.2) in the CP arm. The difference favored CP (1.61; 95% CI 0.44 to 2.77, *p* = 0.009). The mean symptom severity LS scores also improved from 4.5 (SD 3.8) to 3.1 (SD 3.0) in the progesterone arm and from 4.7 (SD 2.8) to 1.9 (SD 1.8) in the CP arm. Although the difference favored CP, it was not statistically significant (1.32; 95% CI −0.25 to 2.89, *p* = 0.095). Complete remission of VLS was observed in 60% of patients with available biopsy in the progesterone arm and 81.3% in the CP arm. The odds ratio (OR) favored CP but was not statistically significant (0.35; 95% CI 0.06 to 2.06, *p* = 0.234). No drug-related serious adverse events occurred during the trial. In summary, the study concludes that after 12 weeks of treatment, topical progesterone 8% ointment is considered inferior to standard therapy with topical CP 0.05% in premenopausal women with VLS who have not received prior treatment. CP demonstrated superior efficacy in improving clinical LS scores and symptom severity and achieving complete remission when compared to topical progesterone [83] (Table 2).

In a small, single-center study, 11 women (aged 18–77) with VLS received CP treatment. The basic therapy involved the progressive application of CP, leading to symptom reduction and improved skin lesions in most patients after three months. Maintenance therapy, lasting 4–12 months, revealed relapses in four women, while five remained symptom-free. Testosterone ointment was applied in 5 women, but 2 had poor tolerance, leading to treatment discontinuation after 1 month and 11 months, respectively. One patient with good tolerance is continuing the therapy. The study emphasizes the chronic nature of VLS, suggesting that while TCSs effectively control lesions in most cases, further exploration of potential causes is crucial [84]. In a more extensive single-center study comprising 102 female patients aged 19–85 (average age 55.08) who had been dealing with VLS for 1–29 years (average duration 4.59 years), the patients either showed no improvement while undergoing CP 0.05% ointment treatment or refused steroid therapy. These patients were enrolled in PDT using 5% 5-ALA in gel form, combined with 2% dimethyl sulfoxide (DMSO). The gel was applied to the vulva, and after 3 h, the affected areas were exposed to a halogenic lamp emitting light in the range of 590–760 nm with a power density of 204 mW/cm^2^, delivering a dose of 120 J/cm^2^ during a 10 min radiation treatment. This PDT was administered once a week for ten weeks. After three months, 87.25% of patients achieved complete or partial remission, while 12.75% showed no improvement with PDT. The most significant vulvoscopic response was the reduction of subepithelial ecchymoses and telangiectasias (78.95%) and decreased erosions and fissures (70.97%). Partial remission of lichenification with hyperkeratosis was noted in 51.61% of cases. The least favorable response was observed in the reduction of atrophic lesions (37.36% of cases). The study showcased good outcomes with PDT, and the treatment was well-tolerated by the participants [85]. In other prospective observational studies, the efficacy of methyl aminolevulinate PDT (MAL-PDT) was assessed to establish a therapeutic algorithm based on disease severity for 33 patients with VLS. Three patients (16.7%) with severe itch and fissurated lesions were considered for MAL-PDT. After careful lesion examination, nine patients underwent MAL-PDT, resulting in complete lesion resolution for all patients. For moderate–severe stages of VLS, MAL-PDT proved to be an effective treatment, providing resolution of lesions in all treated patients [86].

The impact of PDT on the immune status in VLS was explored, with a specific focus on the antinuclear antibody (ANA) profile in a single study from Poland. This investigation involved 100 women with VLS, with or without a concurrent autoimmune disease. All participants underwent 10 cycles of PDT. The study evaluated autoimmune antibodies before and after PDT alongside clinical response assessment in a two-year prospective controlled before-and-after study. Following PDT, a significant reduction in the intensity of symptoms (itching, pruritus, vulvar discomfort) was observed. Post-therapy results showed a partial response in 41% of patients, no symptoms in 51%, and persistent or worsened symptoms in 8%. The most prevalent autoimmune diseases included thyroid disorders, vitiligo, and arthritis. Among patients with an additional autoimmune disease (40 patients) before PDT, 57% had ANA antibodies. The mean ANA level in this group significantly decreased after PDT treatment (261.74 IU/mL before vs. 123.20 IU/mL after treatment). In conclusion, the authors suggest that PDT may impact the immune status of patients with VLS [87].

Using 5-ALA-PDT (85 mW/cm2 and 62.5 J/cm) with green light (540 nm) in patients with VLS presents a notable advantage regarding irradiation tolerance, avoiding the intense pain commonly associated with red light. This observation is supported by a study conducted by Osiecka BJ et al. involving 11 women (aged 30–66) with VLS. The study revealed positive outcomes within 2 months post-PDT, with itching subsiding in 81.8% of the participants. After 4 months, 72.7% experienced no itching, and this figure persisted at 63.6% after 6 months. During the 6-month observation period, only 3 patients (27.27%) reported weak itching, primarily before menstruation, while one woman (9%) complained of moderate itching throughout the entire post-PDT period. It suggests that 5-ALA-PDT with green light is well-tolerated and can effectively alleviate itching in patients with VLS, presenting a promising approach for managing this condition [88].

Mitchell L et al. conducted a prospective, double-blinded, randomized, sham-controlled study to assess the efficacy of fractionated carbon dioxide (CO_2_) laser therapy for VLS. The study included 40 women with confirmed VLS through biopsy, randomized in a 1:1 ratio to receive either five sham laser treatments or five fractionated CO_2_ treatments over 24 weeks. The intention-to-treat (ITT) analysis comprised 37 women (19 fractionated CO_2_, 18 sham), with three excluded due to a lack of post-treatment biopsies. Results showed a 0.20 reduction (improvement) in the histopathology scale score from baseline in the active treatment group (95% CI −1.1, 0.80, *p* = 0.74), while the sham treatment group exhibited a 0.1 increase from baseline (95% CI −0.90, 1.0, *p* = 0.91). The change in the histopathology scale score between the active and sham arms was not statistically significant (95% CI −1.14, 1.06, *p*= 0.76). Therefore, this study concludes that fractionated CO_2_ is not an effective monotherapy for treating VLS [89]. On the contrary, in a recent randomized study involving 20 women with VLS, the feasibility of using fractional CO_2_ laser was compared to the standard treatment with CP 0.05%. Among them, nine underwent CP (N1), involving nightly applications in the first month, followed by applications on alternate nights in the second month, and a consecutive two-day application in the third month. The remaining 11 women received laser therapy (N2), consisting of three sessions with a 30-day interval between sessions. The laser sessions were conducted with specific settings: power 25 watts/Stack 1/time 700 microseconds/Spacing 700 micromillimeters. The evaluation after 3 months of treatment revealed the following results: itching in N2 with a mean (µ) of 9.18 (standard deviation (SD) = 0.87) and in N1 with µ 8.44 (SD = 1.88), *p* = 0.204; dysuria in N2 with µ 8.80 (SD = 2.90) and in N1 with µ 9.33 (SD = 1.66), *p* = 0.283; pain in N2 with µ 9.18 (SD = 0.75) and in N1 with µ 9.00 (SD = 1.66), *p* = 0.187; sexual activity in N2 with µ 6.67 (SD = 2.89) and in N1 with µ 6.50 (SD = 3.70), *p* = 0.682; appearance in N2 with µ 8.82 (SD = 2.40) and in N1 with µ 8.50 (SD = 0.93), *p* = 0.242; satisfaction in N2 with µ 9.82 (SD = 0.40) and in N1 with µ 9.33 (SD = 1.32), *p* = 0.006; difficulty in N2 with µ 2.64 (SD = 0.83) and in N1 with µ 2.56 (SD = 1.03), *p* = 0.615; so this study revealed that fractional CO_2_ laser emerges as a promising therapeutic option, particularly for patients who exhibit minimal or partial responsiveness to CP [90].

In another single-center randomized controlled trial comparing fCO_2_ with CP for patients with VLS, 52 participants were randomized, with 51 completing a 6-month follow-up (N1: fCO_2_ arm—27 patients, mean age 67.6 ± 11.0; N2: CP arm—24 patients, mean age 61.5 ± 8.9). In the ITT analysis, N1 showed significantly greater improvement compared to N2 (N1: −16.83 ± 18.09 vs. N2: −5.92 ± 5.81; *p* = 0.007). Similar results were observed in the per-protocol analysis, with N1 (n = 26) demonstrating greater improvement compared to N2 (n = 19) (N1: −16.46 ± 17.21 vs. N2: −5.79 ± 5.29; *p* = 0.007) and a size effect of −10.66 (95% CI −18.93 to −2.39). Improvement was notable in emotional well-being (N1: −19.63 ± 21.92 vs. N2: −6.77 ± 9.9; *p* = 0.011) and symptom relief (N1: −21.03 ± 22.18 vs. N2: −4.91 ± 11.19; *p* = 0.002), while the function subscore was similar between the groups (N1: −10.65 ± 18.97 vs. N2: −5.30 ± 8.64; *p* = 0.210). At 6 months, 89% of N1 participants rated their symptoms as “better or much better” on the Patient Global Impression of Improvement (PGI-I) compared with 62% of N2 patients (*p* = 0.073). Overall, 58% of the participants were “satisfied or very satisfied” with the Patient Global Impression of Satisfaction (PGI-S); notably, significantly more participants (81%, N1) expressed satisfaction compared with the N2 group (41%, *p* = 0.011) [91].

On the other hand, a recent prospective, randomized, double-blinded, dose-controlled trial indicates that treatment with the micro ablative CO_2_ laser results in a notable enhancement of symptoms associated with VLS. A total of 67 patients with VLS were included in the study and were divided into two groups: the Low Dose Group (LDG) [29 patients, mean age 66.2 (54–80.5)] and the Normal Dose Group (NDG) [34 patients, mean age 67.5 (55–81.5)]. Over the 18-week study period, both groups underwent three laser applications in three consecutive sessions of three weeks, with follow-ups at six and twelve weeks after the first application. Before treatment, the mean Visual Analogue Scale (VAS)-Score was 4.3 (±2.4) in the NDG and 5.1 (±2.6) in the LDG. After 18 weeks, the mean reduction was −2.4 (±2.3) for NDG and −2.7 (±2.8) for LDG. Notably, four patients (two from each group) reported increased pain after treatment. Both groups exhibited significantly lower VAS-Scores 18 weeks after treatment than before therapy (*p* < 0.0001). However, the reduction of symptoms after 18 weeks between NDG and LDG was not statistically significant (*p* = 0.6244) [92].

In a study conducted by Pagano T et al., the effectiveness of rescue fCO_2_ was assessed in women experiencing severe symptoms and sexual dysfunction associated with VLS who did not respond to prolonged ultra-potent TCSs. Among the 40 eligible participants, a notable improvement was observed in vulvar itching (χ^2^ ^[2]^ = 31,182, *p* < 0.001), vulvar dryness (χ^2^ ^[2]^ = 40,364, *p* < 0.001), superficial dyspareunia (χ^2^ ^[2]^ = 37,488, *p* < 0.001), and sensitivity during intercourse (χ^2^ ^[2]^ = 22,143, *p* < 0.001) following two cycles fCO_2_. The pain associated with probe movement and laser application remained low and did not show significant changes post-treatment. Notably, no systemic or local adverse effects were reported during or after the laser treatment. The findings suggest that fCO_2_ is a safe option and may serve as an effective rescue procedure for VLS patients who do not respond to extended ultra-potent TCSs [93].

A total of 40 patients were enrolled and randomly assigned in a 1:1 ratio to either the laser group (N1 = 20, mean age 59, SD = 10) or the control group (N2 = 18, mean age 57, SD = 14) through sealed envelope drawing. Patients in the laser group underwent three Nd:YAG (neodymium-doped yttrium aluminum garnet) laser treatments every 14 days, utilizing a fluence of 90 J/cm^2^, while those in the control group received TCS betamethasone. At the 6-month follow-up, the study compared outcomes between N1 and N2 for various symptoms associated with vulvar conditions. For burning, N1 showed a significant decrease of 4.4 points (95% CI: 2.4–6.5, *p* < 0.001), N2 experienced a reduction of 1.7 points (95% CI: 0.2–3.1, *p* = 0.038), and N1 demonstrated a more significant reduction compared to N2, with an effect size of 3.9 (95% CI: 1.2–6.6, *p* = 0.008); for itching, N1 exhibited a significant decrease of 4.9 points (95% CI: 2.4–7.4, *p* = 0.001), N2 showed a decline of 3.0 points (95% CI: −1.3–7.3, *p* = 0.095), and N1 demonstrated a more significant reduction compared to N2, with an effect size of 3.5 (95% CI: −0.3–7.3, *p* = 0.066); for pain, N1 showed a significant decrease of 5.4 points (95% CI: 3.3–7.5, *p* < 0.001), N2 experienced a decline of 2.0 points (95% CI: −3.0–7.0, *p* = 0.225), and N1 demonstrated a slight reduction compared to N2, with an effect size of 0.6 (95% CI: −4.0–5.1, *p* = 0.793); for overall symptom sum, N1 exhibited a significant decrease of 14.7 points (95% CI: 10.5–18.9, *p* < 0.001), N2 showed a decline of 5.7 points (95% CI: 0.5–10.8, *p* = 0.042), and N1 demonstrated a more significant reduction compared to N2, with an effect size of 7.6 (95% CI: −1.0–16.1, *p* = 0.080). N1 demonstrated substantial improvements in burning, itching, pain, and overall symptom sum compared to N2, with notable effect sizes favoring the laser treatment [94].

A retrospective observational study indicated that focused ultrasound therapy showed positive outcomes for patients with VLS, vulvar lichen simplex chronicus (LSC), and vulvar lichen planus (LP). The study included 85 (LSC), 44 (VLS), and 7 (LP), with an age range 14–75 years old and mean age 41.5 ± 12.0 years. All patients underwent ultrasound therapy (parameters: power 3.5 W, frequency 10 MHz, pulses 1000 Hz) with the following results: VLS, cured: 23 (52.27%), effective: 17 (38.64%), ineffective: 4 (9.09%); LSC, cured: 41 (48.24%), effective 39 (45.88%), ineffective: 5 (5.88%); LP, cured: 4 (57.14%), effective: 3 (42.86%), ineffective: 0. The study concluded that focused ultrasound therapy is an effective therapeutic option for VLS. However, it is essential to note that this is an observational study, and further research, including randomized controlled trials, may be needed to establish the efficacy of focused ultrasound therapy for these skin conditions more conclusively. Additionally, considerations for potential side effects and long-term outcomes should be addressed in future investigations [95].

The complete clearance of VLS remains infrequent among treated patients, potentially impacting their well-being. A recent observational study on a cohort of 101 women with VLS aimed to evaluate whether achieving complete clearance with a TCS (mometasone furoate (MMF) 0.1% ointment for 12 weeks) translates into benefits in terms of patient suffering and quality of life (QoL) impairment. At the completion of treatment, the authors compared patients who achieved clearance in symptoms (Global Subjective Score [GSS] = 0), objective features (Global Objective Score [GOS] = 0), or both with those who experienced a lower degree of improvement based on Pictorial Representation of Illness and Self-Measure (PRISM) and Dermatology Life Quality Index (DLQI) scores. 34 patients (35.8%) achieved GSS = 0, 26 (25.7%) achieved GOS = 0, and 11 (11.5%) achieved clearance of both GSS and GOS. PRISM scores were significantly higher in patients who achieved clearance of symptoms than those who did not, even in cases of substantial (50–99%) GSS improvement from baseline. DLQI scores were lower in patients who achieved clearance of symptoms, signs, or both compared to others. The study concludes that obtaining clearance of VLS corresponds to a significant improvement in the QoL of patients, even when compared to those with substantial but incomplete reductions in symptom and sign scores, making it an ideal therapeutic goal [96]. In another single-center, retrospective, open-label, comparative trial, the efficacy and tolerability of MMF 0.1% ointment were assessed over a 24-week treatment period for VLS in one group (N1, n = 29, aged 42–86 years). The results of this study were retrospectively compared with those achieved using the same glucocorticoid molecule, administered with the same treatment regimen, but over a 12-week duration in another group (N2, n = 32, aged 40–86 years). Three patients (10.3%) dropped out in N1, and four (12.5%) dropped out in N2. The dropout rate did not differ significantly between the two study groups (*p* = 0.99). Results indicated that out of the total participants, 31 (50.8%) achieved GSS = 0, with 16 (55.2%) in N1 and 15 (46.9%) in N2. Additionally, 23 (37.7%) achieved GSS greater than 0, with 10 (34.5%) in N1 and 12 (40.6%) in N2 (*p* = 0.554). Regarding the GOS, 10 (16.4%) achieved GOS = 0, including 4 (13.8%) in N1 and 6 (18.8%) in N2. Furthermore, 44 (72.1%) achieved GOS greater than 0, with 22 (75.9%) in N1 and 22 (68.7%) in N2 (*p* = 0.730). Additionally, 8 (13.1%) achieved both GSS and GOS equal to 0, including 3 (10.3%) in N1 and 5 (15.6%) in N2. Lastly, 46 (73.8%) achieved both GSS and GOS greater than 0, with 23 (79.4%) in N1 and 23 (71.9%) in N2 (*p* = 0.706). The results suggest that a 24-week duration of corticosteroid treatment does not confer significant therapeutic advantages compared to the standard 12-week duration courses concerning clearance of VLS [97]. Also, Corazza M et al. conducted a cross-sectional study in 2020 on the burden of suffering in patients with chronic inflammatory vulvar diseases using PRISM and assessing its concordance with DLQI. The study included 87 LS patients, 13 with lichen simplex chronicus (LSC) and 7 with lichen planus (LP). Median PRISM values (95–120 mm) varied by disease, and median DLQI scores were consistent across the groups. PRISM and DLQI scores did not significantly differ among the three conditions. A moderate correlation was found between PRISM and DLQI (ρ = 0.5455, *p* < 0.001). The GSS was the only variable significantly linked to suffering and quality-of-life impairment. PRISM proved to be a valuable, highly sensitive tool for measuring perceived burden, surpassing DLQI in capturing patient distress [97]. Subsequently, the researchers enrolled 63 patients with VLS from the previous cross-sectional study into a prospective study [98] and underwent a 12-week treatment with a topical MMF 0.1%. Significant changes were observed in both PRISM and DLQI scores post-treatment. PRISM’s median score increased from 85 (55–180) to 180 (90–270) (*p* < 0.0001), while DLQI’s median score decreased from 6 (3–8) to 2 (1–5) (*p* < 0.0001). The coefficient of variation (CV) for PRISM (1.59) was slightly higher than DLQI (1.55). This finding aligns with the moderate correlation between PRISM and DLQI score variations after treatment (ρ = −0.54; *p* < 0.001). Consequently, the study implies that PRISM may be more reliable than DLQI in capturing changes in the disease-related burden post-treatment and accurately quantifying the baseline burden [99].

A prospective cross-sectional study from Australia assessed the QoL in VLS patients undergoing long-term, personalized TCS. It included 204 biopsy-proven VLS patients, comprising 68 new pretreatment (N1) and 136 treated patients on topical corticosteroids for 2 years or more (N2). N2 scored lower in all VQLI components [100], including total score, symptoms, anxiety, activities of daily living, and sexuality (all *p* < 0.001). A higher proportion of N2 achieved minimal impact of VLS on quality of life (98 (72.1%) vs. 8 (11.8%); *p* < 0.001). Mild and reversible adverse effects were observed in 11 patients (8.1%). Partially compliant patients were 12-times more likely to develop scarring progression than fully compliant patients (7 (22.6%) vs. 2 (1.9%); *p* < 0.001). Long-term, individualized TCS is deemed safe and effective in maintaining disease remission and enhancing the VLQI for VLS patients [101].

Kohn JR et al. examined the effects of TCS use on adult women with VLS in their retrospective cohort research. A total of 1525 visits were made by 333 women (62.7 years) (median 6 visits/patient; range, 1–24 visits). In 26% of the visits, patients used fewer TCSs than recommended, 8% did not, and 66% used TCSs precisely as prescribed. In comparison to the absence of steroid use, regular use demonstrated a notable improvement in symptoms (odds ratio [OR] 4.6; 95% confidence interval [CI] 2.2–9.6) and physical examination findings (OR 6.9; 95% CI 2.7–17.6). On the other hand, infrequent steroid use also exhibited positive effects, albeit to a lesser extent (symptoms: OR 2.5; 95% CI 1.2–5.4; physical examination findings: OR 4.2; 95% CI 1.6–11.0). These findings suggest a gradient of impact, with regular use showing the most significant association with improved outcomes, followed by infrequent use. Further, 42% of women reported being inactive sexually at intake due to pain; following TCS, 37% of these women resumed their sexual activity. There was no correlation between changes in sexual activity and steroid adherence. The study underlines the significance of accurate TCS use in improving outcomes for women with VLS. Still, it also draws attention to the shortcomings in the consistent recording of sexual activity, which may have an effect on how quality of life outcomes are evaluated [102].

In another observational study also led by Corazza M., 17 women with VLS (N1, mean age 66.76, SD 11.09) were treated with a combination of MMF 0.1% ointment and tretinoin (T) 0.05% cream, while 15 women with VLS (N2, mean age 63, SD 9.85) received treatment with MMF 0.1% ointment and a cold cream (CC). The study found that combining topical MMF with T did not yield dermoscopic benefits when compared to the same corticosteroid used alone. This suggests that the addition of T to the corticosteroid regimen did not provide an observable advantage in terms of dermoscopic outcomes in VLS patients. Regarding vessels, N1: 64.7% at baseline to 100% after 12 weeks (*p* < 0.001), N2: 100% at baseline and after 12 weeks, and no significant difference in the change between the two groups (*p* = 0.13); whitish areas, N1: 100% at baseline to 58.8% after 12 weeks (*p* < 0.001), N2: 93.3% at baseline to 53.3% after 12 weeks (*p* < 0.001), and no significant difference in the change between the two groups (*p* = 0.71); whitish background, N1: 100% at baseline to 94.1% after 12 weeks (*p* < 0.001), N2: 100% at baseline and after 12 weeks, and no significant difference in the change between the two groups (*p* = 0.09); comedo-like openings, N1: 58.8% at baseline to 17.6% after 12 weeks (*p* < 0.001), N2: 60% at baseline to 40% after 12 weeks (*p* = 0.02), and no significant difference in the change between the two groups (*p* = 0.27); red-purpuric globules or blotches, N1: 35.3% at baseline to 23.5% after 12 weeks (*p* = 0.02), N2: 26.6% at baseline to 13.3% after 12 weeks (*p* = 0.28), and no significant difference in the change between the two groups (*p* = 0.49); scales, N1: 5.8% at baseline to 5.8% after 12 weeks (*p* = 1.00) and N2: 26.6% at baseline to 0% after 12 weeks (*p* = 0.04); gray-blue dots with a peppering pattern, N1: 47% at baseline to 52.9% after 12 weeks (*p* = 0.41) and N2: 33.3% at baseline to 26.6% after 12 weeks (*p* = 0.31) [103].

In a prospective observational study, a single-armed approach without control groups was employed on 64 women diagnosed with VLS (with an average age of 41.5 years). Two intradermal injections of a mixed compound (MB) were administered at a two-week interval. The injected MB compound consisted of methylene blue, dexamethasone powder, ropivacaine, and normal saline, targeting the affected area. Itching severity was self-reported using a VAS, where a score of 10 represented the most severe itching, and 0 indicated no itching. Doctors assessed Squamous Hyperplasia Area Percentage (SHAP) using a vulva surface area scale, dividing it into specific regions. Before treatment, participants reported an itching score of 7.85 ± 1.61. Following treatment, itching scores significantly decreased after 1 month (1.85 ± 2.56) and remained relatively stable in the subsequent follow-ups at 3, 6, 12, and 24 months. Squamous Hyperplasia Area Percentage (SHAP) showed a significant decrease after 1 month of treatment (29.50 ± 19.80 to 28.20 ± 19.31) and continued to decrease over the follow-up period. A recurrence of itching was observed in 21.2% of cases. Complications included vulvar edema, pain, blue staining, numbness, and blue urine. Although some women experienced an increase in itching scores after remission, none surpassed the initial score. In conclusion, the treatment effectively alleviated itching in VLS patients, and SHAP decreased significantly. Recurrence rates were relatively low [104].

**Table 2:** Studies within the last 6 years concerning VLS and the outcomes of personalized conventional therapies (please see references no. [81,82,83,84,85,86,87,88,89,90,91,92,93,94,95,96,97,98,99,100,101,102,103]).

First Author Year Type of Study [Reference]	Studied Population	Results	Conclusions
Krause E 2023 Prospective, randomized, double-blinded, dose-controlled [92]	N1 = 29 women with VLS, LDG (aged 54–80.5) N2 = 34 women with VLS, NDG (aged 55–81.5)	BT: N2 vs. N1 VAS 4.3 (±2.4) vs. 5.1 (±2.6); AT: N2 vs. N1 VAS −2.4 (±2.3) vs. −2.7 (±2.8) AT N1 + N2 vs. BT N1 + N2 *p* < 0.0001	Microablative CO_2_ laser results in a notable enhancement of symptoms associated with VLS; however, the reduction of symptoms after 18 weeks between NDG and LDG was not statistically significant (*p* = 0.6244).
Borghi A 2023 Observational [99]	N = 101 women with VLS treated with mometasone furoate 0.1%	35.8% GSS = 0; 25.7% GOS = 0; 11.5% GSS and GOS = 0	Clearance of VLS corresponds to a significant improvement in the QoL of patients, making it an ideal therapeutic goal.
Salgado HC 2023 Randomized, prospective [90]	N = 20 VLS women; N1 = 11 N treated with tCP; N2 = 9 N treated with fCO_2_	µ(SD) at 3 m - itching: N2 vs. N1, 9.18 (0.87) vs. 8.44 (1.88), *p* = 0.204; - dysuria: N2 vs. N1, 8.80 (2.90) vs. 9.33 (1.66), *p* = 0.283; - pain: N2 vs. N1, 9.18 (0.75) vs. 9.00 (1.66), *p* = 0.187; - sexual activity: N2 vs. N1, 6.67 (2.89) vs. 6.50 (3.70), *p* = 0.682; - appearance: N2 vs. N1, 8.82 (2.40) vs. 8.50 (0.93), *p* = 0.242; - satisfaction: in N2 vs. N1, 9.82 (0.40) vs. 9.33 (1.32), *p* = 0.006; - difficulty: N2 vs. N1, 2.64 (0.83) vs. 2.56 (SD = 1.03), *p* = 0.615	fCO_2_ laser emerges as a promising therapeutic option, particularly for patients who exhibit minimal or partial responsiveness to CP.
García-Souto F 2022 Retrospective, observational [82]	N1 = 62 women with VLS (46.33 ± 2.33 y); N2 = 86 women with ODVA (41.01 ± 1.29 y)	T N1 vs. N2: - ultra-potent tCS: 72.6% vs. 18.6%, *p* < 0.001; - tCI: 9.7% vs. 0%, *p* = 0.005; - physiotherapy: 14.5% vs. 29.1%, *p* = 0.038;	These findings underscore the nuanced T landscape for N1 and N2; furthermore, adjuvant T, such as topical ketoconazole, demonstrated significant disparities between the two groups, emphasizing the need for tailored therapeutic approaches in managing VLS.
Günthert AR 2022 Randomized, double-blinded, 2-armed [83]	N1= 17 VLS receiving tP 8%; N2= 20 VLS receiving tCP 0.05%	- tP arm after 12w 4.6 (SD 2.0) to 4.5 (SD 1.7); - tCP arm after 12w 4.6 (SD 2.8) to 2.9 (SD 2.2); - VLS score N1 vs. N2 (1.61; 95% CI 0.44 to 2.77, *p* = 0.009); - symptom severity VLS N1 vs. N2 (1.32; 95% CI −0.25 to 2.89, *p* = 0.095); - complete remission of VLS: 60% N1; 81.3% N2 N2 vs. N1 OR (0.35; 95% CI 0.06 to 2.06, *p* = 0.234)	tCP superior efficacy in improving clinical VLS scores and symptom severity, as well as in achieving complete remission, when compared to tP.
Mitchell L 2021 Randomized, prospective, double-blinded [89]	N1 = 19 VLS women randomized to 5SLT; N2 = 18 VLS women randomized to 5fCO_2_	N2: 0.20 reduction in HPsS (95% CI −1.1, 0.80, *p* = 0.74); N1: 0.1 increase in HPsS (95% CI −0.90, 1.0, *p* = 0.91)	N1 vs. N2 HPsS (95% CI −1.14, 1.06, *p* = 0.76) not statistically significant.
Corazza M 2021 Retrospective, open-label, comparative [98]	N = 61 VLS women; N1 = 29 N treated 24 w (aged 42–86 y); N2 = 32 N treated 12 w (aged 40–86 y)	- GSS = 0, 50.8% N, N1 vs. N2, 55.2% vs. 46.9%; - GSS greater than 0, 37.7% N, N1 vs. N2, 34.5% vs. 40.6%, *p* = 0.554; - GOS = 0, 16.4% N, N1 vs. N2, 13.8% vs. 18.8%; - GOS greater than 0, 72.1% N, N1 vs. N2, 75.9% vs. 68.7%, *p* = 0.730; - GSS + GOS = 0, 13.1% N, N1 vs. N2, 10.3% vs. 15.6%; - GSS and GOS greater than 0, 73.8% N, N1 vs. N2, 79.4% vs. 71.9%, *p* = 0.706	24 w duration of corticosteroid treatment does not confer significant therapeutic advantages compared to standard 12 w duration courses concerning clearance of VLS.
Burkett LS 2021 Randomized, controlled [91]	N1 = 27 women with VLS randomized in the fCO_2_ arm (mean age 67.6 ± 11.0), N2 = 24 women with VLS randomized in the CP arm (mean age 61.5 ± 8.9)	- ITT: N1 vs. N2, −16.83 ± 18.09 vs. −5.92 ± 5.81 (*p* = 0.007); - per-protocol analysis: N1 vs. N2, −16.46 ± 17.21 vs. −5.79 ± 5.29 (*p* = 0.007), size effect of −10.66 (95% CI −18.93 to −2.39); - emotional well-being: N1 vs. N2, −19.63 ± 21.92 vs. −6.77 ± 9.9 (*p* = 0.011); - symptom relief: N1 vs. N2, −21.03 ± 22.18 vs. −4.91 ± 11.19 (*p* = 0.002); - functional: N1 vs. N2, −10.65 ± 18.97 vs. −5.30 ± 8.64, *p* = 0.210)	At 6 m: - 89% of N1 rated their symptoms as “better or much better” on PGI-I compared to 62% of N2 (*p* = 0.073); - 81% N1 expressed satisfaction vs. N2 (41%, *p* = 0.011).
Wijaya M 2021 Prospective, cross-sectional [101]	N1 = 68 new pretreatment women with VLS; N2 = 136 treated women with VLS > 2 y	- N2 vs. N1, (98 [72.1%] vs. 8 [11.8%]; *p* < 0.001); - risk of scarring in partially compliant N2 vs. fully compliant N2, 7 [22.6%] vs. 2 [1.9%]; *p* < 0.001	Long-term, individualized topical corticosteroid treatment is deemed safe and effective in maintaining disease remission and enhancing the quality of life for VLS patients.
Borghi A 2020 Prospective [99]	N = 63 women with VLS receiving MMF 0.1%, 12 w	- PRISM mean, BT vs. AT, 85 (55–180) vs. 180 (90–270), *p* < 0.0001; - DLQI mean, BT vs. AT, 6 (3–8) vs. 2 (1–5), *p* < 0.0001; - PRISM vs. DLQI, 1.59 vs. 1.55	PRISM may be more reliable than DLQI in capturing changes in disease-related burden post-treatment, as well as accurately quantifying baseline burden.
Pagano T 2020 Prospective, longitudinal [93]	N = 40 women with VLS treated with 2 cycles fCO_2_	- vulvar itching (χ^2^ ^[2]^ = 31,182, *p* < 0.001); - vulvar dryness (χ^2^ ^[2]^ = 40,364, *p* < 0.001); - superficial dyspareunia (χ^2^ ^[2]^ = 37,488, *p* < 0.001); - sensitivity during intercourse (χ^2^ ^[2]^ = 22,143, *p* < 0.001)	fCO_2_ is a safe option and may serve as an effective rescue procedure for VLS patients who do not respond to extended ultra-potent TCSs.
Kohn JR 2020 Prospective, observational [102]	N = 64 women (aged 41.5 ± 13.1 y) with VLS treated with MBmc	- itching score: BT 7.85 ± 1.61; AT 1m 1.85 ± 2.56; AT 3m 1.62 ± 2.59; AT 6m 2.20 ± 3.09; AT 12m 2.33 ± 3.08; AT 24m 2.60 ± 3.26 - SHAP: BT 29.50 ± 19.80; AT 1m 28.20 ± 19.31; AT 3m 23.25 ± 19.44; AT 6m 20.93 ± 20.08; AT 12m 20.13 ± 20.49; AT 24m 20.10 ± 20.50	T with MBmc effectively alleviated itching in VLS patients, and SHAP decreased significantly.
Bizjak Ogrinc U 2019 Randomized, controlled [94]	N1 = 20 women with VLS, mean age 59, SD 10; N2 = 18 women with VLS, mean age 57, SD 14	6 m visit: - burning, N1 −4.4 (95% CI: 2.4–6.5, *p* < 0.001), N2 −1.7 (95% CI: 0.2–3.1, *p* = 0.038), N1 > N2, 3.9 (95% CI: 1.2–6.6, *p* = 0.008); - itching, N1 −4.9 (95% CI: 2.4–7.4, *p* = 0.001), N2 −3.0 (95% CI: −1.3–7.3, *p* = 0.095), N1 > N2, 3.5 (95% CI: −0.3–7.3, *p* = 0.066); - pain, N1 −5.4 points (95% CI: 3.3–7.5, *p* < 0.001), N2 −2.0 (95% CI: −3.0–7.0, *p* = 0.225), N1 > N2, 0.6 (95% CI: −4.0–5.1, *p* = 0.793); - overall symptom sum, N1 −14.7 points (95% CI: 10.5–18.9, *p* < 0.001), N2 - 5.7 points (95% CI: 0.5–10.8, *p* = 0.042), N1 > N2, 7.6 (95% CI: −1.0–16.1, *p* = 0.080)	N1 demonstrated significant improvements in burning, itching, pain, and overall symptom sum compared to N2, with notable effect sizes favoring the laser treatment.
Gajewska M 2018 Longitudinal [84]	N = 11 VLS receiving tCP (aged: 18–77 y)	- 36.36% (4 of N): relapses - 45.45% (5 of N): symptom-free and receiving tT: 80% discontinuation and 20% good tolerance	TCSs effectively control lesions in most cases.
Corazza M 2018 Observational [103]	N1 = 17 women (mean age: 66.76, SD 11.09) with VLS receiving MMF 0.1% + T 0.005%, 12 w; N2 = 15 women (mean age: 63, SD 9.85) with VLS receiving MMF 0.1% + CC, 12 w	- Vessels, N1 BT vs. N1 AT, 64.7% vs. 100% (*p* < 0.001), N2 BT vs. N2 AT, 100%, N1 vs. N2 (*p* = 0.13); - whitish areas, N1 BT vs. N1 AT, 100% vs. 58.8% (*p* < 0.001), N2 BT vs. N2 AT, 93.3% vs. 53.3% (*p* < 0.001), N1 vs. N2 (*p* = 0.71); - whitish background, N1 BT vs. N1 AT, 100% vs. 94.1% (*p* < 0.001), N2 BT vs. N2 AT, 100%, N1 vs. N2 (*p* = 0.09); - comedo-like openings, N1 BT vs. N1 AT, 58.8% vs. 17.6% (*p* < 0.001), N2 BT vs. N2 AT, 60% vs. 40% (*p* = 0.02), N1 vs. N2 (*p* = 0.27); - red-purpuric globules or blotches, N1 BT vs. N1 AT, 35.3% vs. 23.5% (*p* = 0.02), N2 BT vs. N2 AT, 26.6% vs. 13.3% (*p* = 0.28), N1 vs. N2 (*p* = 0.49); - scales, N1 BT vs. N1 AT, 5.8% vs. 5.8% (*p* = 1.00), N2 BT vs. N2 AT, 26.6% vs. 0% (*p* = 0.04); - gray-blue dots with a peppering pattern, N1 BT vs. N1 AT, 47% vs. 52.9% (*p* = 0.41), N2 BT vs. N2 AT, 33.3% vs. 26.6% (*p* = 0.31)	Addition of T to the corticosteroid regimen did not provide an observable advantage in terms of dermoscopic outcomes in VLS patients.
Maździarz A 2017 Longitudinal [85]	N = 102 VLS receiving 5-ALA + 2-DMSO and PDT (aged: 19–85 y)	- Complete/partial remission: 87.25%; - no improvement: 12.75% - reduction of subepithelial ecchymoses and telangiectasias: 78.95% - decrease of erosions and fissures: 70.97%; - partial remission of lichenification with hyperkeratosis: 51.61%; - reduction of atrophic lesions: 37.36%	Favorable outcomes and well-tolerated with PDT.
Olejek A 2017 Longitudinal [87]	N = 100 with VLS receiving PDT N1 = 40 N with cAD N2 = 60 N without aAD N3 = 23 N1 with ANA+	- partial response: 41% N; - no symptoms: 51% N; - worsened symptoms 8% N; - N3: ANA = 261.74 IU/mL before vs. ANA = 123.20 IU/mL after PDT	PDT may have an impact on the immune status of patients with VLS.
Osiecka BJ 2017 Longitudinal [88]	N = 11 women (aged 30–66 y) with VLS receiving 5-ALA-PDT + GL	2 m AT: itching subsiding in 81.8% N; 4 m AT: no itching in 72.7%; 6 m AT: no itching in 63.6%, weak itching 27.27%, moderate itching 9%	PDT + GL is well-tolerated and can effectively alleviate itching in woman with VLS.
Wu C 2017 Retrospective, observational [95]	N1 = 44 women with VLS, treated with FUT; N2 = 85 women with vLSC, treated with FUT; N3 = 7 women with vLP, treated with FUT, mean age 41.5 ± 12.0 y	N1 vs. N2 vs. N3 - cured: 52.27% vs. 48.24% vs. 57.14%; - effective: 38.64% vs. 45.88% vs. 42.86%; - ineffective: 9.09% vs. 5.88% vs. 0	FUT is an effective therapeutic option for VLS.

Abbreviations: N = number of patients; y = years; vs. = versus; VLP = vulvar lichen sclerosus; SD = standard deviation; OR = odds ratio; CI = confidence interval; CV = coefficient of variation; M = males; F = females; GSS = global subjective score; tCP = topical clobetasol propionate; QoL = quality of life; DLQI = dermatology life quality index; PRISM = pictorial representation of illness and self-measure; AT = after treatment; BT = before treatment; LDG = low dose group; NDG = normal dose group; VAS = visual analogue scale; tP = topical progesterone; tT = topical testosterone; tCS = topical corticosteroids; tCP = topical clobetasol propionate; w = weeks; m = months; µ = mean PDT = photodynamic therapy; ALA = aminolevulinic acid; DMSO = dimethyl sulfoxide; SLT = sham laser treatments; fCO_2_ = fractionated carbon dioxide laser; HPsS = histopathology scale score; aAD = additional autoimmune disease; ITT = intention-to-treat; ANA = antinuclear antibody; GSS = global subjective score; GOS = global objective score; MMF = mometasone furoate; GL = green light; CC = cold cream; T = tretinoin; PGI-S = patient global impression of satisfaction; v = visits to the site; ODVA = other dermatoses of the vulvar area; T = treatment; tCI = topical calcineurin inhibitors; FUT = focused ultrasound therapy; vLSC = vulvar lichen simplex chronicus; vLP = vulvar lichen planus; MBmc = methylene blue mixed compound; SHAP = squamous hyperplasia area percentage.

### 3.3. VLS: Platelet-Rich Plasma and Stem Cell Therapy

**Figure 3:**
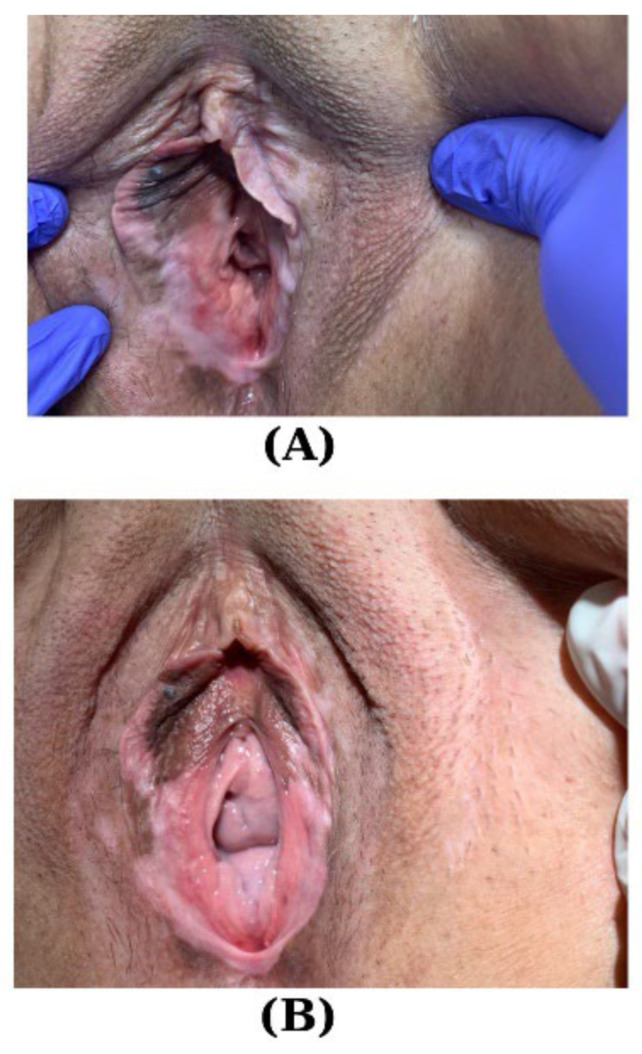
(**A**) A 35-year-old female with obesity, who presented with VLS, presents atrophic papules and macules, hypopigmentation, and fissures; (**B**) image of VLS lesions in the same patient after 3 PRP sessions at four-week intervals—no improvement in clinical appearance and in the symptomatology.

Several observational studies have reported the effectiveness of regenerative therapies that utilize autologous adipose tissue, adipose-derived stem cells, purified vascular stromal fraction, and platelet-rich plasma (PRP) for treating VLS. PRP nourishes cells, enhances the healing process, and promotes the recovery of injured tissue thanks to its elevated levels of growth factors and other bioactive substances [105,106] (Figure 3).

One randomized, double-blinded, placebo-controlled study included 29 women (mean age, 52.6 years) with VLS, randomized into two groups: 10 placebo (saline injections) and 19 autologous PRP (5 mL subdermally and intradermally), where patients were treated with two separate treatments. Of the 19 women receiving PRP, 5 (26.31%) showed improvement in histopathologic inflammation, 10 (52.63%) had no change, and 4 had more inflammation. Of the 10 women receiving placebo, 5 had improvement, 4 had no change, and 1 had more inflammation (*p* = 0.542). The mean difference in the symptoms between initial and final visits was 7.74 for patients receiving PRP and 9.44 for patients receiving the placebo (*p* = 0.654). The study suggests that autologous PRP does not adequately treat VLS, contrasting with some prior studies lacking placebo controls or validated measures [107] (Table 3).

Nevertheless, a single-center, randomized, controlled, pilot trial involved randomly assigning 20 participants to either the nanofat–PRP treatment group (TG = 10) or the 0.05% CP control group (CG = 10). In total, 19 patients remained for the final analysis after one therapy group member withdrew. The findings show notable variations in a number of characteristics. The average relative values of R2 (gross elasticity) and R5 (net elasticity) did not exhibit improvement from the baseline during the 12-month follow-up (R2, *p* = 0.473, and R5, *p* = 0.461). The nanofat–PRP treatment demonstrated effectiveness in ameliorating the clinical manifestations and reducing the symptoms of VLS. Notably, a significant enhancement was observed from the baseline to the 12-month mark in nearly all the scores in the TG. A comprehensive evaluation of the clinical symptoms in the TG revealed noteworthy improvements in the mean scores between the baseline and the 12-month follow-up (itching, *p* = 0.039; pain, *p* = 0.016; burning, *p* = 0.002; dyspareunia, *p* = 0.035; and GSS = 0.006). Conversely, the CG did not display significant improvement in itching (*p* = 0.033939), pain (*p* = 0.250), burning (*p* = 0.477), dyspareunia (*p* = 0.453), and GSS (*p* = 0.469) during the same period. When comparing the 12-month follow-up to the baseline, the evaluation of the clinical indicators revealed a substantial improvement in fissures (*p* = 0.017), stenosis (*p* = 0.043), pallor (*p* = 0.004), and GOS scores (*p* = 0.004) in the TG. Although a decrease in the TG’s erosion score was noted, it was not statistically significant (*p* = 0.094). There was no evidence of improvement regarding the hyperkeratosis, agglutination, and atrophy scores (*p* = 0.500, *p* = 0.250, and *p* = 0.500, respectively). Patients in the CG, on the other hand, did not significantly improve from the baseline state (all *p* > 0.05). Histological analysis at the 12-month follow-up showed that patients in the TG had a substantially decreased number of inflammatory cells (all *p* < 0.05). In contrast, only the number of eosinophils in the CG decreased (*p* = 0.016). The mean number of apoptotic bodies, parakeratosis, hyperkeratosis, hypergranulosis, and acanthosis decreased in the biopsies from the TG, according to the semi-qualitative estimation of direct and indirect chronic lesion markers. However, none of these changes were statistically significant when compared to the CG (all *p* > 0.05), except for the apoptotic bodies (*p* < 0.01). In both groups, there were no appreciable improvements in subepithelial fibrosis or epidermal atrophy (*p* > 0.05). The TG had a significantly improved QoL from the baseline to the 1-year follow-up (79.7 ± 33.2 to 59.7 ± 24.9, *p* = 0.004). Patients in the TG group had better QoL scores at the one-year mark of the study than the CG group after receiving CP treatment (*p* = 0.006). Compared to the topical CP, these results point to a possible benefit of the nanofat–PRP intervention [108].

In an Italian study, the clinical efficacy of local injections of adipose-derived stromal vascular (AD-SVF) was compared with AD-SVF combined with PRP in two randomized groups of patients with genital LS. The study enrolled 40 patients (24 males, 16 females) with a mean age of 43 years (ranging from 18 to 78 years), randomized into two arms with a 1:1 allocation. Each patient underwent two procedures spaced four months apart. The AD-SVF, comprising 15 cc for each patient (with an additional 4 cc of PRP for the second arm), was injected intradermally into the genital area affected by LS. Patients were evaluated one month after each regenerative procedure and six months after the second treatment. Results showed that both groups (AD-SVF and AD-SVF plus PRP) experienced a reduction in symptoms and improvements in skin and mucosal elasticity, hydration, and atrophy. In total, 13 (32.5%) reported a complete disappearance of symptoms, 23 patients (57.5%) exhibited significant improvement, and 4 patients (10%) showed no changes. Notably, two patients (5%) reported a reduction in white lesions. Statistical analysis revealed no significant difference in the mean clinical scores between the AD-SVF and AD-SVF plus PRP groups (AD-SVF = 2.0 ± 0.8; AD-SVF plus PRP = 1.8 ± 1.1, *p* = 0.42). However, AD-SVF-treated patients showed higher scores 2 and 3 than AD-SVF plus PRP (75% vs. 55%). Further analysis based on the disease stage indicated that early-stage patients had a significantly higher clinical score than late-stage patients (early = 2.2 ± 0.8; late = 1.6 ± 1.0, *p* = 0.046). Subgroup analysis showed a significant difference in the AD-SVF plus PRP group based on disease stage (early = 2.6 ± 0.7; late = 1.3 ± 1.0, *p* = 0.011), whereas no differences were observed in the case of AD-SVF treatment (early = 2.0 ± 0.8; late = 2.0 ± 0.9, *p* = 0.77). Additionally, the analysis of male and female patients did not reveal significant differences in the clinical score (F = 2.0 ± 1.1; M = 1.79 ± 0.9, *p* = 0.52). After 6 months of treatment, the overall improvement in the QoL was significant (*p* = 0.0002). The median DLQI value decreased from 5.0 ± 3.9 to 2.32 ± 1.85; AD-SVF alone: DLQI improvement was substantial (*p* = 0.003); AD-SVF plus PRP: DLQI improvement was significant (*p* = 0.014); early-stage disease: AD-SVF plus PRP had better results than AD-SVF alone (*p* = 0.036); late-stage disease: combination therapy (PRP + AD-SVF) had poor outcomes compared to AD-SVF alone (*p* = 0.0083) [109].

**Table 3:** Studies within the last 6 years concerning VLS and the outcomes of innovative treatment modalities (please see references no. [106,107,108]).

First Author Year Type of Study [Reference]	Studied Population	Results	Conclusions
Gutierrez-Ontalvilla P 2022 Prospective, randomized [107]	N = 19 women with VLS, N1 = 9 N randomized nanofat–PRP, N2 = 10 N randomized tCP 0.05%	N: R2 AT *p* = 0.473, R5 AT *p* = 0.461; N1 AT vs. N2 AT - itching, *p* = 0.039 vs. *p* = 0.033939; - pain, *p* = 0.016 vs. *p* = 0.250; - burning, *p* = 0.002 vs. *p* = 0.477; - dyspareunia, *p* = 0.035 vs. *p* = 0.453; - GSS, *p* = 0.006 vs. *p* = 0.469; N1 AT: fissures (*p* = 0.017), stenosis (*p* = 0.043), pallor (*p* = 0.004), GOS scores (*p* = 0.004), erosion (*p* = 0.094), hyperkeratosis (*p* = 0.500), agglutination (*p* = 0.250), atrophy (*p* = 0.500); - N2 AT: all clinical signs (*p* > 0.05); - HP: number of inflammatory cells: N1 AT (all *p* < 0.05), N2 AT only eosinophils decreased (*p* = 0.016); - QoL AT: N1 vs. N2, *p* = 0.004 vs. *p* = 0.006	In comparison to tCP, these results point to a possible benefit of the nanofat–PRP intervention.
Tedesco M 2020 Longitudinal [109]	N = 40 patients with GLS (24 M, 16 F; aged 18–78 y), N1 = 20 N randomized to AD-SVF arm, N2 = 20 N randomized to AD-SVF + PRP	- Mean clinical scores: N1 vs. N2, 2.0 ± 0.8 vs. 1.8 ± 1.1, *p* = 0.42; - early N vs. late N, 2.2 ± 0.8 vs. 1.6 ± 1.0, *p* = 0.046; - early N2 vs. late N2, 2.6 ± 0.7 vs. 1.3 ± 1.0, *p* = 0.011; - early N1 vs. late N1, 2.0 ± 0.8 vs. 2.0 ± 0.9, *p* = 0.77; - F N vs. M N, 2.0 ± 1.1 vs. 1.79 ± 0.9, *p* = 0.52; - QoL after 6 m: *p* = 0.0002; - median DLQI BT vs. AT, 5.0 ± 3.9 vs. 2.32 ± 1.85; - N1 DLQI: *p* = 0.003; - N2 DLQI: *p* = 0.014; - early-stage disease N2 vs. N1: *p* = 0.036; - late-stage disease N2 vs. N1: *p* = 0.0083.	Both groups N1 and N2 experienced a reduction in symptoms and improvements in skin and mucosal elasticity, hydration, and atrophy: 32.5% complete disappearance of symptoms; 57.5% significant improvement; 10% no changes; 5% reduction in white lesions. No significant difference in the mean clinical scores between N1 and N2.
Goldstein AT 2019 Randomized, double-blinded, placebo-controlled [108]	N = 29 women with VLS (mean age 52.6); N1 = 19 N randomized in PRP arm; N2 = 10 N randomized in placebo arm	- -HP: N1 vs. N2, N1: 26.31% improvement, 52.63% no change, 21.05% more inflammation; N2: 50% improvement, 40% no change, 10 more inflammation (*p*= 0.542); - -Symptoms: N1 vs. N2, 7.74 vs. 9.44 (*p* =0.654)	PRP does not adequately treat VLS.

Abbreviations: N = number of patients; y = years; vs. = versus; VLP = vulvar lichen sclerosus; SD = standard deviation; GLS = genital lichen sclerosus; PRP = platelet-rich plasma; AD-SVF = adipose-derived stromal vascular fraction; M = males; F = females; R2 = gross elasticity; R5 = net elasticity; GSS = global subjective score; tCP = topical clobetasol propionate; QoL = quality of life; DLQI = dermatology life quality index; AT = after treatment; BT = before treatment.

## 4. Discussion

The lack of a clear cure for VLS presents a problem in the medical field, mainly due to the uncertain etiology of the disease. The optimal treatment strategy seeks to alleviate symptoms, reverse signs, and avoid further structural abnormalities, such as the potential formation of malignancies. Due to the continuous and ongoing development of VLS, it is advisable to provide treatment for all instances, regardless of symptoms. In order to effectively treat VLS, it is essential to target two main pathophysiological events: (1) auto-immunogenic pathways that cause inflammation and oxidative stress and (2) the formation of fibrous tissue. Treatment methods should prioritize targeting either one or both of these pathways. At present, there is no universally endorsed treatment for VLS. Multiple therapy alternatives are accessible, and the selection is contingent upon various criteria. These criteria encompass disease-related elements such as length and severity, clinical features, and symptoms.

Furthermore, factors such as the patient’s age, expectations, and the effect on their QoL are pivotal in establishing the most appropriate course of therapy. Treatment choices vary depending on whether the therapy is intended for acute or maintenance purposes. The main objective of treatment is to achieve not only the remission of symptoms but also the long-term maintenance of a condition free from symptoms, which is accomplished through successful therapy [110].

### 4.1. Dynamics of Sexual Hormones in VLS Individuals

This review rigorously analyzes the current literature to assess the reliability of evidence about the development and long-term treatment methods for girls during puberty and adult women, with a focus on innovative and novel techniques. Given the possible difficulties that may arise from the extended use of strong TCSs, it is essential to comprehend how VLS develops during adolescence. This understanding is critical for deciding whether aggressive or more cautious treatment approaches are necessary.

As previously mentioned, VLS is most frequent in prepubertal and postmenopausal women, suggesting hormonal causes. One study identified low androgen receptor levels in VLS lesions [111], while another found low 5-a reductase levels [23]. Despite the increased occurrence of VLS in hypo-estrogenic females and the efficiency of estrogen treatment in children, this condition was previously treated with testosterone or progesterone, not estrogen [112,113,114]. Research on the role of estrogen and androgen receptors in the development of various disorders has been contentious. Some investigations have shown no receptors for estrogen (ERs) or androgen (ARs) in normal tissues, despite the vulva and vagina being estrogen and androgen target targets [115,116]; other researchers have found ARs and ERs in normal vulvae [25]. Biochemical investigations show poor estrogen- and androgen-binding sites in vulval biopsies of VLS patients [117]. The expression of ER-α and ARs in vulvar lichen sclerosus have demonstrated a lack of AR expression in most instances. Only the ‘fibrovascular’ layer of the vulva showed a correlation between ER-β and Ki-67 expression and ER-α expression. In the LS vulva, ERa expression was lacking in the ‘fibrovascular’ layer, while ER-b expression was absent in normal tissues but abundantly elevated in diseased vulvas [111].

The existing literature on lichen sclerosus in girls and teenagers is limited [118]. The signs and symptoms of VLS typically continue throughout puberty, and most cases show permanent architectural alterations or scarring. The extent of these changes varies, ranging 40–58% in some instances [80,81]. The approach employed is descriptive; however, the study is constrained by a restricted number of subjects. Consequently, it is imperative to conduct larger-scale investigations in order to obtain more robust results.

### 4.2. Insights into VLS Treatment Approaches: Examining the Evolution of Approaches and the Lessons from Varied Therapeutic Strategies over Time

The appropriate treatment of VLS is also supported by the risk of malignant transformation. In a population-based study assessing the malignant potential of VLS, findings revealed 812 cases of cancer among 7,616 patients during the follow-up period (Standardized Incidence Ratio [SIR]: 1.13, 95% CI 1.05–1.21). Notably, LS was linked to an elevated risk of vulvar cancer (182 cases, SIR: 33.6, 95% CI 28.9–38.6) and vaginal cancer (4 cases, SIR: 3.69, 95% CI 1.01–9.44). Conversely, the risk of cancer affecting the uterine cervix and lung was significantly decreased. These findings underscore the association between VLS and an increased risk of vulvar and vaginal cancer, emphasizing the importance of considering these risks in the care of women with this diagnosis [32,119]. Spekreijse JJ et al. conducted a study to assess the exact risk and occurrence rate of SCC in individuals with anogenital LS. The study revealed that the absolute risk of developing SCC is higher in LS patients, with a range of 0.21–3.88% for women and 0.00–0.91% for men, compared to the known figures for the general population, which is 0.002% for women [120]. VLS is linked to a higher likelihood of developing SCC, particularly as individuals get older. Ultrapotent TCSs appear to decrease this risk [121]. A retrospective analysis of medical records from a dermatogynecology practice in Australia examined the rate of SCC recurrence in patients with VLS who underwent long-term TCS therapy over a period of 12 years. The study found that patients who consistently followed the TCS therapy had a recurrence rate of 27% for SCC, which is lower than the reported 5-year recurrence rates of 44% to 47% [122].

An autoimmune origin has been suggested for LS due to its connection with autoimmune disorders and the presence of organ-specific antibodies in circulation [31,123]. Cooper SM et al. conducted a study involving 190 women exhibiting typical features of adult-onset LS of the vulva, with 112 women serving as the control group; some specific autoimmune disorders were significantly more frequent in the LS cohort compared with controls, including thyroid disease, alopecia areata, pernicious anemia, and morphea. Serum autoantibody assays were conducted in 190 LS patients and 112 controls, revealing the presence of one or more serum antibodies in 21% of the LS cohort and 20% of controls [31]. Female patients with LS exhibit a higher frequency of associated autoimmune diseases, particularly autoimmune thyroid disease, along with the presence of circulating autoantibodies. In contrast, these characteristics are absent in male patients with LS [124]. This association highlights the potential autoimmune involvement in the etiology of LS. It emphasizes the necessity of doing a comprehensive examination of the patient’s medical background and conducting a thorough study in the event of identifying another related autoimmune disorder.

Topical CP 0.05% ointment or cream is considered the most effective treatment for VLS. This conclusion is based on extensive use over a long period and numerous randomized controlled trials [50,125,126]. Substantial evidence supports the effectiveness of 0.1% MMF in treating VLS [127,128,129]. The duration required to establish remission of VLS varies, but based on the authors’ observations, it typically takes approximately 3–6 months of consistent and powerful TCS treatment. The limitation of the majority of the published research is its limited duration, typically spanning only 3–6 months. There is a scarcity of long-term observational studies that include sufficient individuals who have received treatment. Most articles have a recorded observation period of up to 3 years, the most extended duration [130]. There are three exceptions to note. Firstly, there is descriptive cohort research from the UK, with an average follow-up duration of 66 months. Secondly, a long-term study from France was conducted prospectively over a period of 10 years. Lastly, there is a study from Australia that was also conducted prospectively but for 8 years. These exceptions are referenced as [22,51,77]. The study above, which involved 507 women, provides the most compelling data to validate the knowledge held by seasoned practitioners. While TCS effectively initiates remission, it does not offer a cure for VLS. In addition, the French investigation documented a recurrence rate of 84% if the treatment was discontinued. These studies indicate that the treatment has the potential to alter the progression of the disease, thereby increasing the likelihood of developing cancer and minimizing the formation of scars. The Australian observational study presented highly persuasive evidence. There is evidence supporting this claim [22]. The subjects of this investigation were individuals who were receiving medical treatment. The items were assessed on a regular basis, often every 3 to 6 months, to determine their effectiveness. The strength of the TCS was gradually reduced to a moderate to mild level through titration for ongoing therapeutic maintenance. This was attainable for approximately 75% of the individuals receiving medical treatment. Subsequent recovery necessitated further treatment involving additional sessions. TCSs are commonly employed in VLS and can lead to local side effects including atrophy, striae, and purpura when used excessively. Adverse effects that affect the entire body as a result of using steroids are uncommon and primarily associated with taking them orally. These factors involve the disruption of the hypothalamic–pituitary–adrenal axis, resulting in the development of Cushing syndrome. Factors that increase the likelihood of developing Cushing syndrome as a result of using TCSs include age, skin condition, strength of the steroid, treated area, treatment duration, and the use of occlusive dressings. While VLS patients get treatment according to the existing standards, the prolonged application of high potency TCSs on a mucosal surface does indeed raise the chance of developing Cushing syndrome. Nevertheless, although several females of various age groups are subjected to an identical treatment, there is a lack of documented instances of Cushing syndrome, with only a limited number of cases recorded in the literature [131].

In the past, topical testosterone was commonly used to treat VLS. However, it is no longer considered a viable option due to the availability of more effective treatments and the potential for androgenization in girls [132,133,134]. The absence of androgen receptors in VLS is an active keratinocyte phenotype proposed. These results could help explain why treating with testosterone does not work. Furthermore, no discernible change in symptoms was observed in relation to menstruation, pregnancy, oral contraceptives, or hormone replacement medication [135]. Also, progesterone and estrogen have little effect on the course of the disease. Similarly, there is no difference in the expression of nuclear estrogen receptor and progesterone receptor between patients with VLS and controls [136]. However, using hormones in VLS has yielded inconsistent results among studies. The effectiveness of testosterone and progesterone local therapy in vaginal meshing surgery was assessed in a survey by Zarcone et al. [136]. Out of 65 women diagnosed with vulvar lichen sclerosus, 26 (68%) of 38 women responded completely to testosterone treatment; 18 (66.6%) of the 27 women who received progesterone treatment showed a complete response [136]. Topical estrogen has no purpose other than to alleviate hypo-estrogenic atrophy in post-menopausal women. While managing post-menopausal women with VLS, it is crucial to consider this issue, but it does not serve as a targeted treatment. Nevertheless, the persistent discomfort caused by vaginal dryness and fragility, which are symptoms of the genitourinary syndrome of menopause, might complicate the evaluation of therapy effectiveness for concurrent VLS. Therefore, it is crucial to address both symptoms simultaneously.

As demonstrated in the described studies [85,86,87], in the early stages, PDT can be a viable alternative well tolerated by patients, reducing itching and dryness and improving mucosal texture. The utilization of MAL-PDT proves to be an effective treatment in moderate to severe stages of LSA. Eleven studies involving 337 women with VLS provided evidence that PDT effectively reduces symptoms associated with the disease. However, the evaluation of clinical improvement in lesion appearance remains unclear, and the understanding of this subject is somewhat limited. Additional observational studies involving a large number of patients are necessary in order to clarify the effectiveness of PDT [137]. The reported outcomes of VLS in response to treatment with the CO_2_ laser range from quite favorable to unsatisfactory [138,139]. One possible reason for this contrast could be the partial removal of the affected tissue. While not typically used as the initial treatment, it can be successful in bringing about relief from symptoms and remission of the condition in challenging patients. Moreover, this technique is considered safe and poses minimal risk to the patient. The lack of success in laser treatment in some studies [89] may be attributed to the failure to select the right clinical endpoint accurately.

The gradual degeneration that defines the clinical progression of VLS might result in an abnormal alteration of the standard structure of the anogenital region. The disease’s inflammatory nature is evidenced by the elevated expression of IFN-γ, TNF-α, and IL1α and a significant restructuring of the fibrillin, collagen, and elastin arrangement in the affected tissue [140,141]. Applying ultrapotent TCSs as a standard treatment effectively improves the primary symptoms. However, long-term persistence of anatomical damage, such as atrophy and scarring, is observed [22,48,128]. In 2010, Casabona et al. were the first to document the utilization of homologous fat grafting and PRP in treating VLS. This approach aims to minimize atrophy and scarring while enhancing skin tropism [106]. Subsequently, many organizations documented an effective therapy for LS patients through adipose tissue transplantation, fat grafting supplemented with SVF, or pure adipose-derived stem cells [105,142,143,144]. The adipose tissue possesses a significant therapeutic capacity due to its high concentration of pluripotent mesenchymal stem cells (MSCs), endothelial progenitor cells, T cells, B cells, mast cells, and adipose-resident macrophages. Additionally, it contains a wide range of bioactive secretory factors, collectively known as AD-SVF; MSCs located in AD-SVF had an immunomodulatory effect on immune cell reactions through their interaction with dendritic cells, T lymphocytes, and natural killer cells [145,146]. The subsequent impact involves the suppression of fibrosis and the promotion of healing, resulting in beneficial outcomes in the restructuring of the extracellular matrix [147,148]. The rationale behind combining PRP with fatty tissue is rooted in the notion that PRP has the potential to enhance the viability of fat grafts [149]. Furthermore, PRP regulates the immune system and extracellular production, two conditions in which VLS changes. In spite of the studied properties of PRP, the included studies in the analysis highlight contradictory results [107,109], emphasizing the need for longer-term observational studies with larger populations to standardize this treatment. On the other hand, all studies involving nanofat–PRP and AD-SVF [108,109] show promising results and should be considered in the management of patients with VLS to prevent or improve architectural disorganization. Assurance in the impact of the effectiveness of AD-SVF and PRP treatment is constrained by their intrinsic limitations, similar to those involving the CO_2_ laser. These observational studies lack essential elements like sample characteristics, control/comparison groups, placebo, grouping, randomized allocation, and unbiased treatment evaluation.

The literature reports a diagnostic delay of VLS, which can have a detrimental impact on the prognosis [1,79]. This delay may be caused by various factors, such as a lack of awareness regarding the anatomy of the female external genitalia, not only among women [150,151] but also among clinicians [152]. Disseminating information regarding normal external female genital anatomy and vulvar self-examination (VSE) is crucial in order to promote early detection of vulvar malignancies and other abnormalities. Therefore, additional endeavors are required to ensure that women are well-informed and confident in performing VSE [151]. In contrast, a study carried out in Italy assessed the knowledge of obstetrics and gynecology residents and revealed a substantial requirement for enhancing their understanding of VLS [152]. Additional endeavors are required to disseminate information regarding VLS and implement extensive training programs.

## 5. Conclusions

Advances in disease formation and progression hold promise for improved treatment of VLS, bringing us one step closer to minimally invasive options that could prevent and treat architectural dystrophy. Emerging treatments, including PRP and stem cell therapy, and energy-based lasers like fractional CO_2_ and ND-Yag are being investigated to identify more effective VLS treatments than ultrapotent topical corticosteroids. However, more research is needed to assess the efficacy and safety of these new medicines. Topical clobetasol 0.05% ointment daily for 4–12 weeks remains the gold standard for treating VLS and preventing scarring and malignancy.

## Figures and Tables

**Table 1:** Studies within the last 6 years concerning VLS and the evolution amid pubertal hormonal changes (please see references no. [80,81]).

First Author Year Type of Study [Reference]	Studied Population	Results	Conclusions
Boero V 2023 Observational retrospective study [81]	N = 31 pVLP (mean age: 6.3 years; SD ± 2.58)	N at re-examination: - 12.9% VLS - 12.9% itching - 12.9% burning - 12.9% vulvar discomfort - 6.5% dyspareunia - 9.7% vulvar dryness - 61.3% no relevant symptoms	At re-examination: 58.1% were considered still affected by VLS, 16.1% achieved complete remission, and 25.8% were asymptomatic despite clinical signs.
Winfrey OK 2022 Retrospective [80]	N1 = 141 premenarchal women with VLS N2 = 36 postmenarchal women with VLS N3 = 26 premenarchal women with VLS followed through the pubertal transition	38.5% in the N3 continued to experience VLS symptoms - vulvar itching N1 vs. N2 = 70.2% vs. 52.8%; *p* = 0.048 - vulvar bleeding N1 vs. N2 = 26.2% vs. 5.6%; *p* = 0.008 - bowel symptoms N1 vs. N2 = 16.3% vs. 0%; *p* = 0.009 - subepithelial hemorrhages N1 vs. N2 = 24.8% vs. 5.6%; *p* = 0.01 - clitoral adhesions N2 vs. N1 = 25.0% vs. 4.3%; *p* < 0.0001 - loss of labia minora N2 vs. N1 = 47.2% vs. 2.1%; *p* < 0.0001	VLS can persist after menarche in approximately 40% of adolescents and may also initially develop in postmenarchal adolescents, with differences in initial symptoms and examination findings based on menarchal status.

Abbreviations: N = number of patients; y = years; vs. = versus; pVLP = pediatric vulvar lichen sclerosus; SD = standard deviation.

## Data Availability

Data sharing not applicable.

## Abbreviations

AD-SVF	Adipose-derived stromal vascular fraction
CC	Cold cream
CP	Clobetasol propionate
CV	Coefficient of variation
DLQI	Dermatology life quality index
GOS	Global objective score
GSS	Global subjective score
ITT	Intention-to-treat
LDG	Low dose group
LP	Lichen planus
LS	Lichen sclerosus
LSC	Lichen simplex chronicus
MMF	Mometasone furoate
Nd:YAG	Neodymium-doped yttrium aluminum garnet
NDG	Normal dose group
PRISM	Pictorial representation of illness and self-measure
PRP	Platelet-rich plasma
QoL	Quality of life
SCC	Squamous cell carcinoma
SCT	Stem cell therapy
SHAP	Squamous hyperplasia area percentage
T	Tretinoin
TCS	Potent topical corticosteroids
UV	Ultraviolet radiation
VAS	Visual analogue scale
VLS	Vulvar lichen sclerosus
VSE	Vulvar self-examination
VQLI	Vulvar quality of life index
